# Burkitt’s Lymphoma and B-Cell Lymphoma Unclassifiable With Features Intermediate Between Diffuse Large B-Cell Lymphoma and Burkitt’s Lymphoma in Patients With HIV: Outcomes in a South African Public Hospital

**DOI:** 10.1200/JGO.2015.002378

**Published:** 2016-08-31

**Authors:** Gerhard Sissolak, Matthew Seftel, Thomas S. Uldrick, Tonya M. Esterhuizen, Nooroudien Mohamed, Danie Kotze

**Affiliations:** **Gerhard Sissolak**, **Tonya M. Esterhuizen**, **Nooroudien Mohamed**, and **Danie Kotze**, Tygerberg Academic Hospital and Stellenbosch University, Cape Town, South Africa; **Matthew Seftel**, University of Manitoba, Winnipeg, Manitoba, Canada; and **Thomas S. Uldrick**, National Cancer Institute, Bethesda, MD.

## Abstract

**Purpose:**

Burkitt’s lymphoma (BL) is a common HIV-associated lymphoma in South Africa. B-cell lymphoma unclassifiable with features intermediate between diffuse large B-cell lymphoma and Burkitt’s lymphoma (BL/DLBCL) also occurs in HIV infection. Outcomes of HIV-infected patients with BL or BL/DLBCL in a resource-constrained setting are not defined.

**Methods:**

We performed a retrospective study of HIV-positive patients with BL or BL/DLBCL treated from 2004 to 2012 with curative intent at a publically funded academic medical center in South Africa. Differences between BL and BL/DLBCL, survival outcomes, and factors associated with survival were analyzed.

**Results:**

There were 35 patients with either HIV-associated BL (24) or BL/DLBCL (11) who met study criteria. Median CD4^+^ T-lymphocyte count at lymphoma diagnosis was 188 cells/μL (range, 10 to 535 cells/μL). Patients with BL/DLBCL were significantly older and had less bone marrow involvement and lower baseline serum lactase dehydrogenase than patients with BL. Eighty-nine percent of patients presented with advanced disease, and 25% had baseline CNS involvement. Chemotherapy regimens consisted of cytoreduction with low-dose cyclophosphamide, vincristine, and prednisone followed by induction with vincristine, methotrexate, cyclophosphamide, doxorubicin and prednisone (LMB 86; 57%); hyperfractionated cyclophosphamide, vincristine, doxorubicin, dexamethasone, methotrexate, and cytarabine (hyper-CVAD; 20%); cyclophosphamide, doxorubicin, vincristine, and prednisone and high-dose methotrexate with leucovorin rescue on day 10 with accompanying prophylactic IT chemotherapy (Stanford regimen; 14%); and cyclophosphamide, doxorubicin, vincristine, and prednisone (CHOP-like; 9%) regimens. Twenty-three patients received CNS treatment or prophylaxis, and 31 received concurrent combination antiretroviral therapy. Two-year overall survival was 38% (95% CI, 22% to 54%) and 2-year event-free survival was 23% (95% CI, 11% to 38%), with no difference between histologic subtypes. Common causes of death were infection (41%) and CNS disease progression or systemic relapse (41%).

**Conclusion:**

Cure of HIV-associated BL and BL/DLBCL with intensive regimens is possible in resource-limited settings, but lower toxicity regimens, improved CNS prophylaxis, and increased resources for supportive care are required.

## INTRODUCTION

Burkitt’s lymphoma (BL) is an aggressive non-Hodgkin lymphoma characterized by a c-Myc translocation. HIV substantially increases BL risk. In addition, Burkitt-like lymphoma,^1^ which is currently classified as B-cell lymphoma unclassifiable with features intermediate between diffuse large B-cell lymphoma and Burkitt’s lymphoma (BL/DLBCL) may occur with increased frequency in people with HIV. Cyclophosphamide-based protocols for endemic (HIV-negative) BL in African children have been published^2,3^; however, BL/DLBCL outcomes have not been well evaluated in the setting of HIV. Therapeutic trials for HIV-associated BL have been conducted in developed countries^4-6^ and are based on multiagent anthracycline-based regimens that require substantial supportive care and monitoring. There is no standard approach to HIV-associated BL or BL/DLBCL in sub-Saharan Africa.

Tygerberg Hospital in Cape Town, South Africa, serves a low socioeconomic population^4^ with estimated 16% HIV seroprevalence.^4^ In this setting, the proportion of non-Hodgkin lymphomas attributed to HIV increased from 5% in 2002 to 37% in 2009,^7^ BL composed 35% of lymphomas in HIV-positive individuals, and more than 80% of BL in South Africa is HIV-associated.^7,8^ Before combination antiretroviral therapy (cART) became available and intensive chemotherapy regimens were developed, patients with HIV-related BL had a poor prognosis, with only 7% overall survival (OS) at 4 years.^9^ In resource-rich settings, high-intensity chemotherapy regimens and cART have improved OS in HIV-associated BL with 2-year OS ranging from 47% to 100%.^10-13^ HIV-positive patients treated with full-intensity chemotherapy regimens in the cART era have OS comparable to that of HIV-negative patients.^10,11,13-15^ However, implementation of curative-intent regimens for aggressive HIV-associated lymphomas remains an important public health challenge in South Africa. We previously showed that with available cART, treatment of AIDS-associated DLBCL with cyclophosphamide, doxorubicin, vincristine, and prednisone (CHOP) leads to outcomes comparable to those in developed countries that use the same regimen.^16^ However, treatment outcomes for HIV-associated BL and BL/DLBCL have not been defined in sub-Saharan Africa, where resource limitations and burden of HIV-related complications may have an impact on survival.

## METHODS

We conducted a retrospective analysis of HIV-positive patients with BL or BL/DLBCL treated at Tygerberg Hospital. Case ascertainment and clinical information were derived from the National Health Laboratories electronic database supplemented by hospital records. Approval was obtained from the local institutional review board, and the study complies with the principles of the Helsinki Declaration.

### Patient Selection

We identified patients through an electronic database search. HIV-seropositive adults 18 years of age or older with BL, Burkitt-like lymphoma, or BL/DLBCL who received high-dose chemotherapy from January 2004 to June 2012 were included. The starting date for inclusion was based on initiation of broad-scale cART roll-out in South Africa. Patients with missing clinical records, diagnostic uncertainty, or no follow-up at our center, and patients who were not receiving curative-intent chemotherapy were excluded.

### Histopathologic Diagnosis

**Fig 1:**
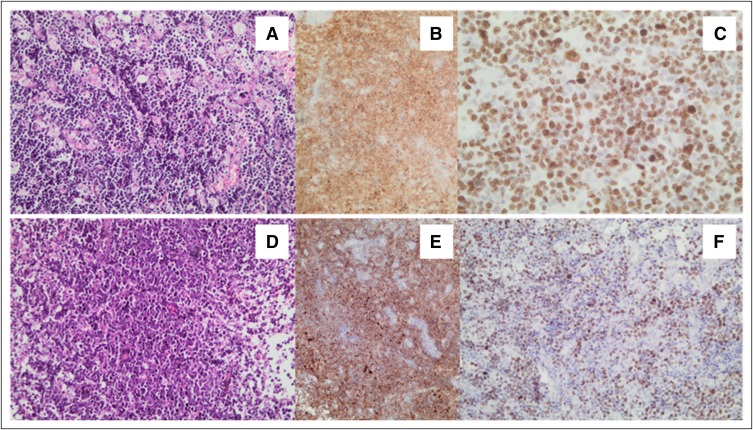
**** Morphologic and immunophenotypic features of Burkitt’s lymphoma (BL) and B-cell lymphoma unclassifiable with features intermediate between diffuse large B-cell lymphoma and Burkitt’s lymphoma (BL/DLBCL). (A) Classic BL with starry sky appearance and monomorphic population of medium-size cells (hematoxylin and eosin; original magnification, ×200). (B) CD10 immunohistochemistry shows diffuse positive staining of lymphoma cells (original magnification, ×200). (C) Ki-67 proliferation index of 100% (original magnification, ×400). (D) BL/DLBCL with similar starry sky appearance and monomorphic population of medium-size cells (hematoxylin and eosin; original magnification, ×200). (E) CD10 immunohistochemistry shows similar diffuse positive staining of lymphoma cells (original magnification, ×200). (F) Ki-67 proliferation index measures lower (< 80%) in BL/DLBCL (original magnification, ×200).

We used WHO 2008 classification criteria,^17^ which recommends that pathologic specimens be examined by two pathologists. In cases of discordance, consensus was reached through discussion at a multiheaded microscope. BL criteria included the following: morphology—medium-size cells and paracentric small nucleoli with a rim of deep basophilic cytoplasm and characteristic starry background; immunophenotype—positivity for CD20, strong expression of germinal center markers (CD10, BCL6), and Ki-67 proliferation index of greater than 95% together with a negative BCL2; genetic features—presence of c-myc rearrangements (8q24) and/or translocation (8;14) fusion genes. Patients who had the typical BL features but also had overlapping features were classified as BL/DLBCL category per WHO 2008 criteria. The criteria included the presence of atypical morphology (eg, the presence of large cells with large nucleoli, more than is acceptable for BL), atypical immunophenotype (eg, bcl-2 expression, MUM-1 expression, Ki-67 < 90%), or atypical genetic features (negative c-myc rearrangement or positive myc rearrangement with BCL2 positivity on immunohistochemistry; Fig 1). Because of resource limitations during the study period, not all participants had molecular confirmation.

### Staging

The Ann Arbor staging system was used.^18^ Baseline assessments included clinical evaluation, bone marrow biopsy, cerebrospinal fluid (CSF) cytopathology, computerized tomography (CT) scan of the chest, abdomen, and pelvis, and brain CT if clinically indicated.^9^ [^18^F]-Fluorodeoxyglucose positron emission tomography was not used.^19,20^ Patients were considered to have CNS involvement if BL cells were noted in cytopathologic evaluation of the CSF, if spinal cord compression was clinically observed, if cranial nerve palsy was present, or if there was an intracranial space-occupying lesion.^14^ Leukemic presentation of BL was defined by predominance of bone marrow or peripheral blood involvement in the absence of significant nodal disease.^17^ Date of death was confirmed by death certificate. Cause of death was based on clinical information. Disease progression or relapse was determined by biopsies or CSF cytopathology and was supplemented by CT or magnetic resonance imaging studies.

### Therapy

Three protocols were used: LMB (Lymphome Malins de Burkitt) 86, hyper-CVAD, and Stanford chemotherapy.^6,9,12,21^ Patients with CNS involvement or leukemic presentation generally received hyper-CVAD or LMB 86. Hyperfractionated cyclophosphamide, vincristine, doxorubicin, dexamethasone, methotrexate, and cytarabine (hyper-CVAD) was accompanied by prophylactic intrathecal (IT) chemotherapy.^3^ IT chemoprophylaxis (methotrexate 12 mg, cytarabine 30 mg, and dexamethasone 1 mg) was given at every cycle of chemotherapy to all patients with either documented or high risk of CNS involvement. LMB 86 consists of cytoreduction with low-dose cyclophosphamide, vincristine, and prednisone followed by induction with vincristine, methotrexate, cyclophosphamide, doxorubicin and prednisone. Subsequent consolidation cycles with etoposide and cytarabine were followed by maintenance cycles containing lower dosages of previously used agents and prophylactic IT chemotherapy.^6,12^ The Stanford regimen consisted of cyclophosphamide, doxorubicin, vincristine, and prednisone and high-dose methotrexate with leucovorin rescue on day 10 with accompanying prophylactic IT chemotherapy.^6^ CHOP-like regimens were used in BL/DLBCL patients with poor performance status or CD4^+^ T-cell counts < 100 cells per µL. In patients with a left ventricular ejection fraction < 45%, doxorubicin was substituted by mitoxantrone (CNOP) to limit cardiotoxicity. Response to therapy was defined as complete remission (CR), partial remission, stable disease, or progressive disease using Cheson criteria.^19^

Interventions to minimize infective complications included antiseptic mouthwash (chlorhexidine gluconate), antifungals (fluconazole), and prophylaxis for pneumonia (trimethoprim-sulfamethoxazole). Prophylactic antibacterials during neutropenia and myeloid growth factors for primary or secondary prophylaxis were not used. Tumor lysis syndrome was treated or prevented by aggressive fluid hydration, urinary alkalinization, and allopurinol. Patients with relapsed or refractory lymphoma were treated with second-line chemotherapy if functional status permitted.

### HIV Management

HIV clinics managed cART by following national guidelines.^22^ The majority of patients were initiated with or maintained by receiving cART during chemotherapy. From 2004 to 2010, the preferred regimen was nevirapine combined with lamivudine and stavudine. From 2010 to 2012, the preferred regimen was efavirenz combined with tenofovir and lamivudine. Guidelines for second-line regimens usually included ritonavir-boosted lopinavir. Individual-level cART medication data were not available. South African monitoring guidelines included HIV viral load and CD4^+^ counts at the start of cART, after 4 months, and thereafter every 12 months.

### Statistical Analysis

Baseline characteristics of patients with BL and BL/DLBCL were compared. Continuous variables were evaluated by using *t* test or Mann-Whitney *U* test; categorical variables were investigated with likelihood ratio χ^2^ tests or Fisher’s exact test. Response rates were evaluated at 6 months. Time-to-event analysis was performed by using Kaplan-Meier analyses to estimate the time from lymphoma diagnosis until death (OS), at which time patients lost to follow-up were censored. In addition to clinical follow-up, patients were identified by national identity numbers, and a search for each patient’s status (alive or dead) was performed by using the Department of Home Affairs register. Follow-up time was truncated at 2 years. We evaluated event-free survival (EFS), defined as a composite of disease progression, death, or loss to follow-up, with data for patients without the event censored at the end of each follow-up time point.^18^ Histologic subtype, baseline CNS involvement, lactase dehydrogenase (LDH), hemoglobin, age, CD4^+^ T-cell count less than 100 cells per µL, lymphoma regimen, baseline cART, cART era (2004-2009 *v* 2010-2012), and acute renal failure were evaluated as risk factors for 2-year OS by using the log-rank test. Two-sided *P* values < .05 were considered statistically significant. STATA, Release 13 (STATA, College Station, TX) was used for analysis.

## RESULTS

### Case Ascertainment

An electronic database search yielded 54 potential patients. Thirty-five patients remained after exclusion, some of whom had no curative-intent chemotherapy (five), missing clinical data (two), duplication as a result of name change (two), HIV not confirmed (three), BL or BL/DLBCL diagnosis not pathologically confirmed (six), and date of diagnosis prior to 2004 (one). BL was diagnosed in 24 patients (78%), including six with a leukemic presentation. BL/DLBCL was diagnosed in 11 patients (32%). The patients with HIV-associated BL in this series represented 92% of all adult BL cases during this time period.

### Patient Characteristics

Median age was 38 years (range, 17 to 53 years), and 60% were female. Twenty-six patients (74%) presented with stage IV lymphoma and nine with CNS involvement. Median CD4^+^ T-lymphocyte count was 188 cells per µL (range, 10 to 535 cells per µL). Eighteen patients (55%) had a CD4^+^ count < 200 cells per µL, and 6 patients (17%) had a CD4^+^ count < 100 cells per µL. Nine (25%) were receiving cART at time of diagnosis, and seven had an undetectable HIV viral load. Twenty-two (63%) were started on cART after lymphoma diagnosis. Four were not started on cART because they were critically ill, and curative-intent lymphoma therapeutics were initiated with the goal of subsequent introduction of cART after initial lymphoma control. Other than HIV, 13 had comorbid conditions, including acute renal impairment (n = 4), chronic hepatitis B (four of 18 tested), deep vein thrombosis (n = 3), pulmonary tuberculosis (n = 2), cryptococcal meningitis (n = 1), and Guillain-Barré syndrome (n = 1).

Pretreatment characteristics of the patients with BL or BL/DLBCL were compared (Table 1). Differences between groups in age, LDH at presentation, and bone marrow involvement were statistically significant. Median CD4^+^ count was not different between groups (*P* = .9). Twelve of 24 patients with BL had confirmed t(8;14), and 12 patients with BL were diagnosed on the basis of morphologic immunohistochemical criteria. Molecular studies were performed in eight of 11 patients with BL/DLBCL, and they showed the following abnormalities: monosomy 8 (n = 1), trisomy 8 (n = 1), t(8;14) (n = 1), no t(8;14) (n = 4), and an extra copy of c-Myc without t(8;14) rearrangement (n = 1).

**Table 1:**
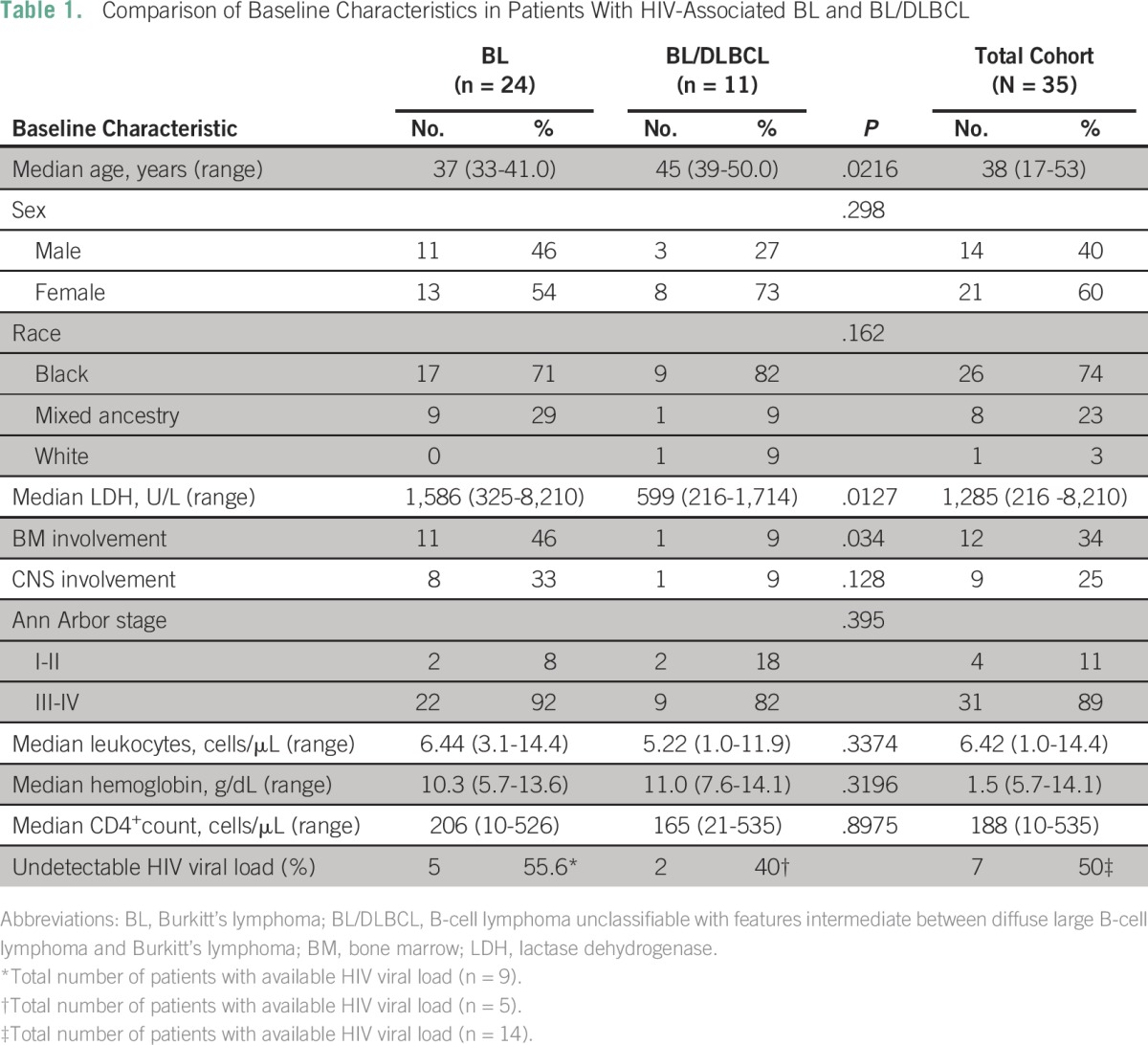
**** Comparison of Baseline Characteristics in Patients With HIV-Associated BL and BL/DLBCL

### Treatment Outcomes

Twenty patients (57%) received LMB 86, seven (20%) hyper-CVAD, five (14%) Stanford regimen, and three (9%) CHOP or CHOP-like regimens (Table 2). For the nine patients with CNS disease, seven received LMB 86 and two received hyper-CVAD. Patients with BL/DLBCL received therapy similar to that for patients with BL apart from three patients with BL/DLBCL who received CHOP-like regimens. Two patients (one with BL and one with BL/DLBCL) were treated with second-line therapy. The most common adverse event during chemotherapy was grade 3 to 4 infection (56%).

**Table 2:**
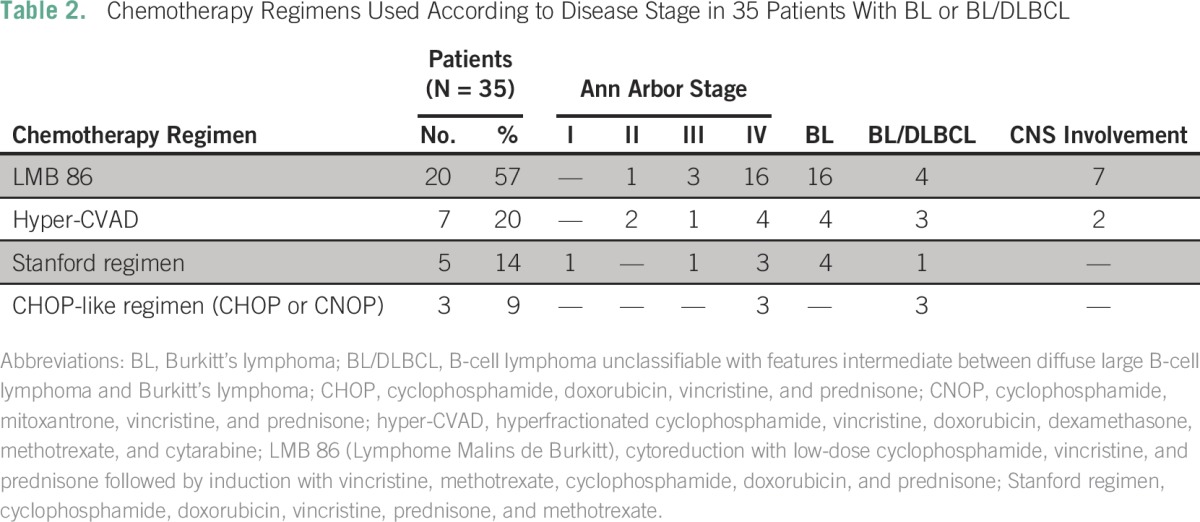
**** Chemotherapy Regimens Used According to Disease Stage in 35 Patients With BL or BL/DLBCL

At 6 months, 10 patients (29%) achieved a CR and seven (20%) achieved a partial remission, whereas three (9%) had progressive disease, and 15 (43%) died. Of three patients with BL/DLBCL treated with CHOP-like regimens, one achieved CR. No patients achieved CR with second-line therapy.

**Fig 2:**
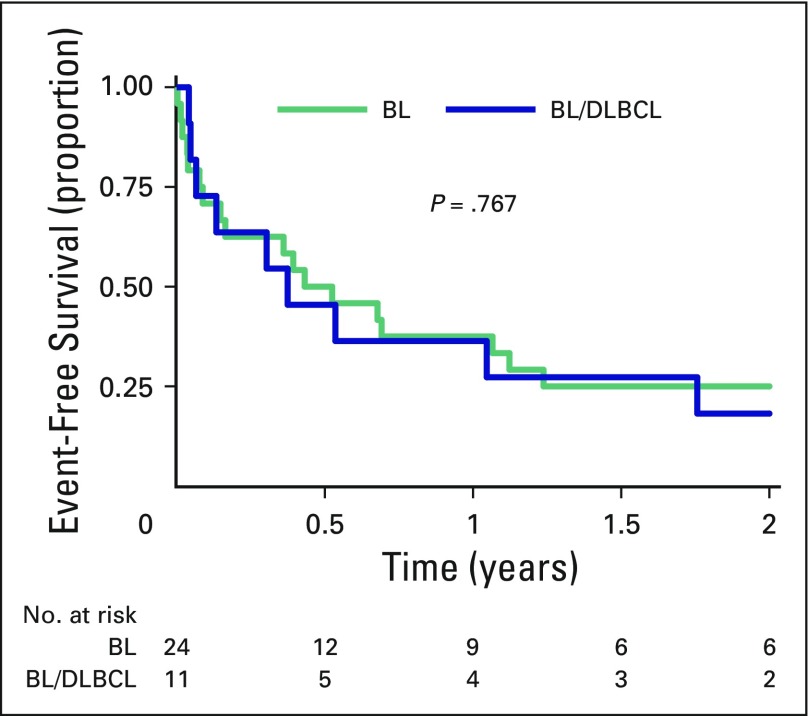
**** Comparison of 2-year event-free survival between patients with HIV-associated Burkitt’s lymphoma (BL) and those with B-cell lymphoma unclassifiable with features intermediate between diffuse large B-cell lymphoma and Burkitt’s lymphoma (BL/DLBCL). Two-year event-free survival was 25% (95% CI, 10% to 43%) in patients with BL and 18% (95% CI, 3% to 44%) in patients with BL/DLBCL.

Six patients were lost to follow-up but were not noted to be deceased in national Department of Home Affairs records. Two-year EFS was 23% (95% CI, 11% to 38%): 25% (95% CI, 10% to 43%) for BL and 18% (95% CI, 3% to 44%) for BL/DLBCL, with no significant differences between groups (*P* = .77; Fig 2).

**Fig 3:**
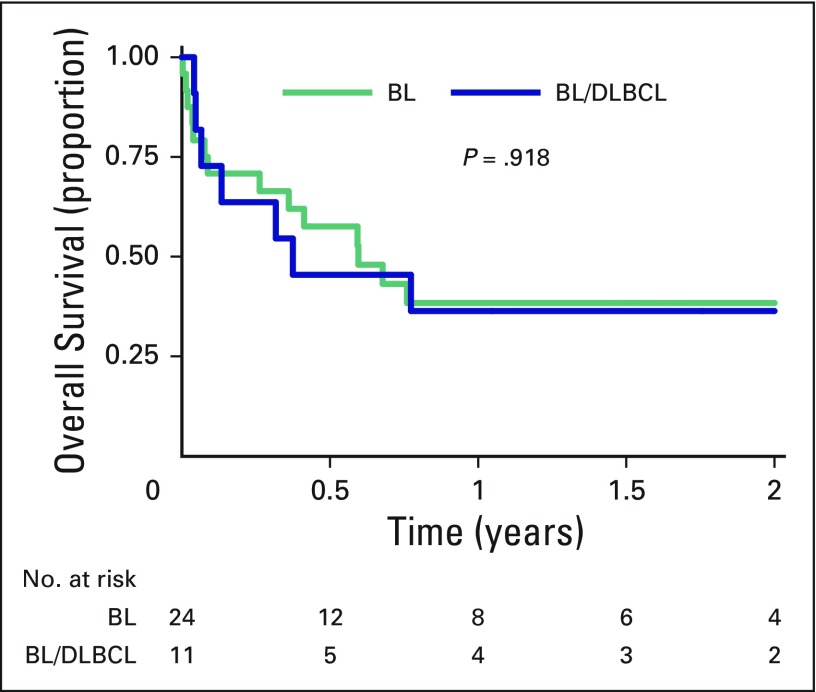
**** Comparison of 2-year overall survival between patients with HIV-associated Burkitt’s lymphoma (BL) and those with B-cell lymphoma unclassifiable with features intermediate between diffuse large B-cell lymphoma and Burkitt’s lymphoma (BL/DLBCL). Two-year overall survival was 38% (95% CI, 19% to 58%) in patients with BL and 36% (95% CI, 11% to 63%) in patients with BL/DLBCL.

**Fig 4:**
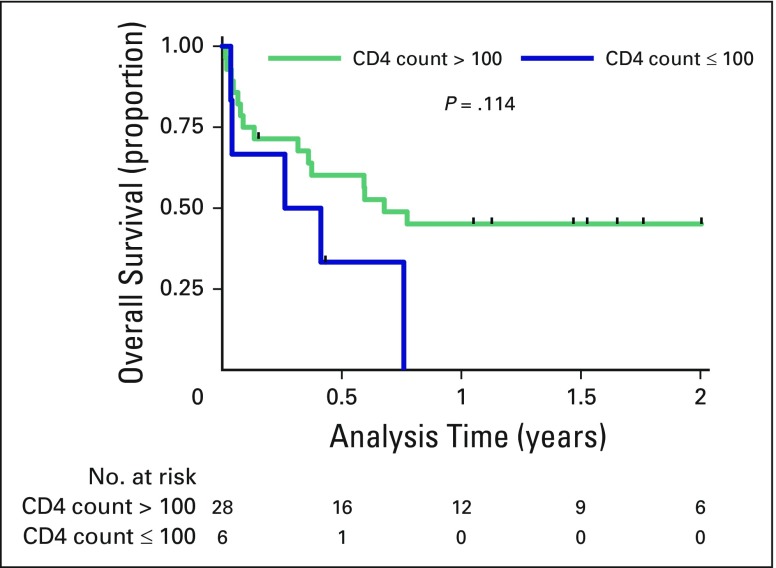
**** Overall survival in HIV-positive patients with baseline CD4^+^ T-cell counts of > 100 cells/μL or < 100 cells/μL. At 24 months, overall survival was 45.1% (95% CI, 26.1% to 62.4%) in patients with > 100 cells/μL and 0% in patients with < 100 cells/μL.

Two-year OS was 38% (95% CI, 22% to 54%) and did not differ between lymphoma subtypes (*P* = .92), with 38% (95% CI, 19% to 58%) 2-year OS in patients with BL and 36% (95% CI, 11% to 63%) 2-year OS in patients with BL/DLBCL (Fig 3). OS was also similar between treatment regimens. Causes of death by year 2 included infection (nine), CNS relapse or progression (seven), other comorbidities (one), and unknown causes (five). No patient with a baseline CD4^+^ count < 100 cells per µL had long-term survival (Fig 4), although this did not reach statistical significance (*P* = .11). Administration of cART was significantly associated with OS (hazard ratio, 0.19; *P* = .008), whereas baseline age, bone marrow disease, CNS involvement, LDH, acute renal failure, baseline cART, and cART administered during the cART era were not.

## DISCUSSION

This analysis of HIV-associated BL and BL/DLBCL provides important observations regarding curative approaches to these lymphomas in a high-HIV-prevalent, resource-limited setting. The predominance of black women reflects the national demographics of HIV prevalence.^23^ The high percentage of BL attributed to HIV (92%) is comparable to that observed in Johannesburg,^8^ which confirms that public health efforts to address BL in South Africa must address HIV. Despite potential differences in the underlying patient populations, the average age (38 years) and median CD4^+^ count (206 cells per µL) is similar to that in international studies of HIV-associated BL.^9,10,13,14,24,25^

We identified some instructive limitations in our study. First, not all cases had c-myc molecular confirmation, which was related to cost constraints and was reflective of the reality of public health oncology care in South Africa. Improved resources for diagnostics are critically important, especially for curable cancers. Second, HIV monitoring was generally followed at community health centers, and improved methods for linking HIV and cancer-related outcomes are needed. In addition, limitations in follow-up may lead to a bias of survival estimates. To address this, we also analyzed EFS, including loss to follow-up as an event. Although EFS estimates were disappointingly low, a small proportion of our patients appear to have durable periods of disease control. Improved resources and methods to ascertain long-term follow-up in oncology patients in resource-limited settings is required.

One strength of this study was our ability to compare BL to BL/DLBCL in people with HIV. BL/DLBCL is a genetically heterogeneous entity and, as noted in other series, not all patients have demonstrable c-Myc abnormalities.^1^ In our study, there were clinical differences between patients with BL/DLBCL and those with BL with respect to age, LDH, and bone marrow involvement. Patients did not differ significantly with respect to CD4^+^ T-cell count, CNS involvement, sex, or disease stage. Higher LDH levels among patients with BL likely represent a higher proliferative rate. Differences in age warrant further epidemiologic assessment and suggest additional biologic differences. Outcomes of patients with BL/DLBCL were similar to those with BL in this series. Apart from three patients who received CHOP-like regimens, the patients with BL/DLBCL in this series received BL-type regimens. Of the three patients who received CHOP-like regimens, only one patient who also received methotrexate obtained a CR. Although frequently treated in the same manner as BL, the optimal regimen for HIV-associated BL/DLBCL requires additional research.^26^

Patients who presented with advanced disease were common, with 89% presenting with stage III or IV disease compared with HIV-positive patients who were enrolled in other international prospective studies (42% to 74% stage III and IV).^9,10,14,24,25^ In addition, eight patients with suspected BL from histology specimens from community-based referral hospitals never arrived at our center for confirmation and staging. Together, these data highlight the need for more efficient oncology referral. Although the optimal timing of initiation of cART in patients with HIV-associated lymphoma is unknown, initiation of chemotherapy in HIV-associated BL and BL/DLBCL is urgent, and oncology referrals should not be delayed while awaiting cART initiation.

Our approach to treating HIV-associated lymphoma was to use concurrent cART and nonnucleoside reverse transcript inhibitor–based regimens that were supported by the public health sector and initiated as soon as feasible. Importantly, patients generally did not receive ritonavir, a drug that inhibits metabolism of many chemotherapy drugs and can increase the risk of adverse events such as profound cytopenias, infectious complications, or neuropathy. Although the majority of HIV-positive patients were not receiving cART at diagnosis, cART was initiated soon after diagnosis. As administered, cART was associated with significantly improved OS. However, a major limitation of this finding is that some patients did not survive long enough despite chemotherapy to start cART. In addition, therapeutic response to cART could not be evaluated because of a lack of available follow-up viral loads.

The best international comparator for OS in patients with HIV-associated BL receiving similar therapy is a prospective study of the LMB regimen^12^ in HIV-positive patients with advanced disease. Although extrapolation of our findings is limited by small sample size and confounding factors, our 2-year OS of 38% (95% CI, 22% to 54%) is comparable to the 2-year OS of 47% (95% CI, 34% to 59%) in the French study,^12^ despite substantial comorbidity in our patients. These data are encouraging and suggest that intensive regimens are feasible in resource-limited settings despite more limited supportive care. Importantly, most deaths in our study population occurred in the first 2 months after starting chemotherapy, and survival reached a plateau 1 year after initiation of therapy.^12,14,25,27^ One contributor to high mortality was profound baseline immunosuppression (CD4 count < 100 cells per µL), with a trend toward poorer OS compared with patients with CD4^+^ counts of ≥ 100 cells per µL (Fig 4). A similar trend in OS between these two groups (*P* = .10) was noted in our previous evaluation of AIDS-related DLBCL.^16^ However, low CD4^+^ counts are a decreasingly important factor in determining OS for patients with aggressive HIV-associated lymphomas in the era of contemporary cART in the United States and Europe,^28^ and monitoring of trends in resource-limited settings remains important because good outcomes may still be achievable in this high-risk patient population. Addition of rituximab to established regimens may further improve outcomes,^13,24^ although access to rituximab remains limited for HIV-infected patients in sub-Saharan Africa.

Treatment of BL requires appropriate initial risk stratification and optimized supportive care. Although we demonstrated that cure of HIV-associated BL and BL/DLBCL is feasible in South Africa, observations from this study demonstrate areas that need improvement. In our patients, most deaths as a result of infection were likely related to myelosuppression. To attain the goals of improved toxicity monitoring and decreased infection-related complications, further development of less intensive protocols, increased availability of myeloid growth factors, investment in infrastructure for administration of chemotherapy, and improved isolation facilities may improve outcomes. Later deaths were largely related to CNS relapse or progression, suggesting that improved approaches to initial CNS disease detection, treatment, and prophylaxis are required.^29,30^ There is much optimism in the field of BL therapy, and excellent responses appear feasible in HIV-infected patients with BL treated with a relatively low-intensity therapy consisting of dose-adjusted infusional etoposide, vincristine, doxorubicin, prednisone, cyclophosphamide, and rituximab (EPOCH-R).^13^ Because patients can potentially be cured, development of improved capacity to treat patients in South Africa with HIV-associated BL and BL/DLBCL, as well as evaluation of regimens that are feasible in the South African public health sector and other resource-limited settings are urgently needed.
